# Remote Excitation of Neuronal Circuits Using Low-Intensity, Low-Frequency Ultrasound

**DOI:** 10.1371/journal.pone.0003511

**Published:** 2008-10-29

**Authors:** William J. Tyler, Yusuf Tufail, Michael Finsterwald, Monica L. Tauchmann, Emily J. Olson, Cassondra Majestic

**Affiliations:** 1 School of Life Sciences, Arizona State University, Tempe, Arizona, United States of America; 2 Harrington Department of Bioengineering, Arizona State University, Tempe, Arizona, United States of America; 3 Array Therapeutic, Scottsdale, Arizona, United States of America; Max-Planck-Institut fuer Neurobiologie, Germany

## Abstract

Possessing the ability to noninvasively elicit brain circuit activity yields immense experimental and therapeutic power. Most currently employed neurostimulation methods rely on the somewhat invasive use of stimulating electrodes or photon-emitting devices. Due to its ability to noninvasively propagate through bone and other tissues in a focused manner, the implementation of ultrasound (US) represents a compelling alternative approach to current neuromodulation strategies. Here, we investigated the influence of low-intensity, low-frequency ultrasound (LILFU) on neuronal activity. By transmitting US waveforms through hippocampal slice cultures and *ex vivo* mouse brains, we determined LILFU is capable of remotely and noninvasively exciting neurons and network activity. Our results illustrate that LILFU can stimulate electrical activity in neurons by activating voltage-gated sodium channels, as well as voltage-gated calcium channels. The LILFU-induced changes in neuronal activity were sufficient to trigger SNARE-mediated exocytosis and synaptic transmission in hippocampal circuits. Because LILFU can stimulate electrical activity and calcium signaling in neurons as well as central synaptic transmission we conclude US provides a powerful tool for remotely modulating brain circuit activity.

## Introduction

Neuromodulation techniques such as deep brain stimulation (DBS) and repetitive transcranial magnetic stimulation (rTMS) have gained widespread attention due to their therapeutic utility in managing numerous neurological/psychiatric diseases [1]. The field of neural control has recently made significant advances by demonstrations of millisecond optical control of individual neurons and synapses in intact brain circuits [2]. Ultrasound (US) as a means of exciting [3] and reversibly suppressing [4] neuronal activity was shown to be effective on a gross level several decades ago. Since then however, explorations into the use of US as a neurostimulation tool have been relatively sparse. The focus has instead been on employing more traditional approaches such as pharmacological, electrical, magnetic, and photonic stimulation of neuronal circuits.

Coupling its ability to interact with biological tissues [5] and its noninvasive transmission through skull bone and other biological tissues in a focused manner [6]–[8], US holds promise as a potentially powerful neurostimulation tool [9], [10], which may be capable of replacing currently invasive DBS strategies. Ultrasound can produce bioeffects by acting through thermal and/or non-thermal mechanisms as it propagates through tissues in pulsed or continuous waveforms [5], [11]–[13]. Therapeutic US can be broadly characterized as low-power/low-intensity or high-power/high-intensity [5]. High-intensity focused ultrasound (HIFU) used in the thermal ablation of tissue implements peak power levels often exceeding 1000 W/cm^2^, whereas non-thermal therapeutic effects of US have been well described at power levels ranging from 30–500 mW/cm^2^ [5], [11]–[13].

Modulation of ionic conductance produced by adiabatic processes as US propagates rapidly and transiently through cellular membranes may alter the activity of individual neurons due to the elastic nature of lipid bilayers and the spring-like mechanics of many transmembrane protein channels. In partial support of this hypothesis, low-power US has been shown to influence the membrane conductance of frog skin epidermis [12]. In addition, US exposure can induce a reversible increase in the internal Ca^2+^ concentration of fibroblasts [14]. In rat thymocytes, stimulation with US can modulate K^+^ influx and efflux [15]. Interestingly, many voltage-gated ion channels, as well as neurotransmitter receptors possess mechanosensitive properties that render their gating kinetics sensitive to transient changes in lipid bilayer tension [16], [17]. Whether or not ion channels can be modulated by US in neurons has remained unknown. Several investigations have demonstrated however that US modulates neuronal activity by enhancing and/or suppressing the amplitudes and/or conduction velocities of evoked nerve potentials [3], [4], [18]–[24].

In a pioneering study, Fry and colleagues (1950) first demonstrated US is capable of modulating neuronal activity by reporting the temporary suppression of spontaneous activity following US transmission through crayfish ventral nerve cords [24]. Transmitting US through the lateral geniculate nucleus of intact cats, Fry and colleagues (1958) demonstrated that high-power US reversibly suppressed light-evoked potentials recorded in the visual cortex [4]. Rinaldi and colleagues (1991) demonstrated that 2.5 to 15 min irradiation of hippocampal slices with 0.75 MHz US (temporal average intensity; I_TA_ ∼80 W/cm^2^), significantly reduces the amplitude of evoked potentials in CA1 pyramidal neurons. In the dentate gyrus of hippocampal slices, focused US pulses have been shown to both enhance and suppress electrically evoked field potentials [21]. In cat saphenous nerve bundles it has been demonstrated that focused US is capable of differentially effecting Aδ- and C-fibers depending on the intensity and duration of US irradiation [23]. In excised frog sciatic nerve bundles, Tsui and colleagues (2005) reported that a temporal average intensity of 1 W/cm^2^ continuous wave (5 min) US (3.5 MHz) increased the amplitude of compound action potentials (CAP), while both 2 and 3 W/cm^2^ intensities decreased CAP amplitudes. Mihran and colleagues (1990) also reported differential excitatory and inhibitory effects of US on frog sciatic CAPs using relatively short irradiation times by delivering 500 µs US pulses (2.0–7.0 MHz) with peak intensities ranging from 100–800 W/cm^2^. Direct activation of the cat auditory nerve has been achieved *in vivo* using 5-MHz US pulses (68 µsec; ∼30 W/cm^2^) [22]. In human subjects, focused US pulses have been shown to activate deep nerve structures in the hand by differentially producing tactile, thermal, and pain sensations [3].

Although numerous intriguing studies examining the influence of US on neuronal activity have been conducted, these previous investigations have implemented high-intensity US, which can destroy nervous tissue. Thus, we decided to investigate the influence of low-intensity ultrasound on neuronal activity. Most of the prior investigations examining the effect of US on neuronal activity also used high-frequency US (>1 MHz; for exceptions see [3], [20], [21]), which has larger attenuation coefficients compared to lower frequency ultrasound. Medical diagnostic US typically operates from 1 to 15 MHz while therapeutic US is usually conducted using acoustic frequencies around 1 MHz [11]. We chose to pursue our investigations here using low-frequency US (0.44–0.67 MHz) since both mathematical models and experimental data indicate the optimal gain between transcranial transmission and brain absorption for US is ∼0.60–0.70 MHz [25], [26]. Detailed cellular investigations into the influence of US on neuronal activity are lacking and the mechanisms underlying US modulation of neuronal activity remain unknown. By optically monitoring changes in ionic conductance in individual neurons and synaptic transmission from individual release sites we investigated the influence of low-intensity, low-frequency ultrasound (LILFU) on central nervous system activity.

## Results

### LILFU activates voltage-gated sodium channels in neurons

**Figure 1:**
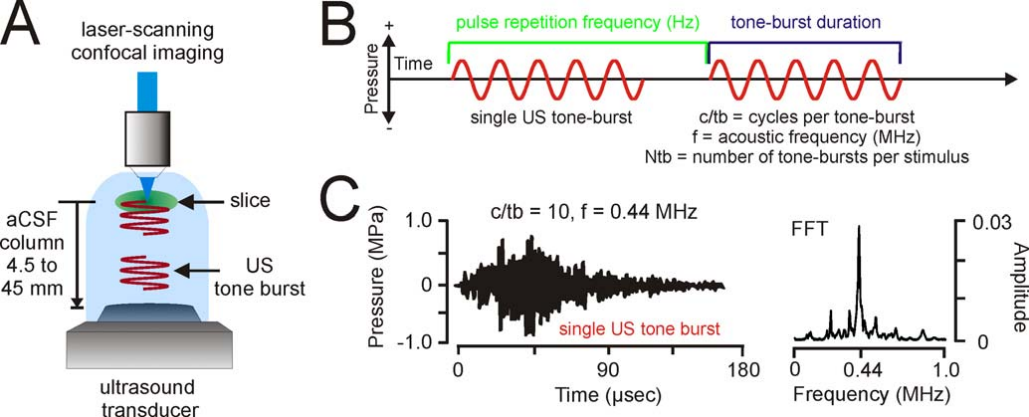
Generation and propagation of LILFU waveforms through neuronal tissue. (A) General experimental configuration implemented to transmit LILFU waveforms through slice cultures while optically monitoring neuronal activity. (B) Graphical illustration of some of the variables involved in constructing LILFU waveforms. These variables include acoustic frequency (f), the number of acoustic cycles per tone burst (c/tb), tone burst duration (TBD), pulse repetition frequency (PRF), and number of tone bursts per stimulus (Ntb). (C) Acoustic pressure wave (*left*) produced by a typical US tone burst consisting of 10 acoustic cycles at f = 0.44 MHz and FFT of this US tone burst (*right*). For the construction of our primary US stimulus waveform (LILFU-1), we used a linearly sweeping PRF by repeating the illustrated tone burst from 0–100 Hz over a 5 sec period.

We transmitted LILFU waveforms through hippocampal slice cultures from remotely positioned tissue-matched piezoelectric (PZT) transducers (Figure 1A). We constructed LILFU waveforms by repeating US tone bursts at variable pulse repetition frequencies (Figure 1B). Measured using a needle hydrophone a points in the recording chamber, which corresponded to slice positions (Figure S1), the predominant LILFU waveform used in our studies (LILFU-1) had a pulse average intensity (I_PA_) of 2.9 W/cm^2^ and a temporal average intensity (I_TA_) of 23 mW/cm^2^. Figure 1C illustrates a typical pressure wave obtained for a single US tone burst used in the construction of LILFU-1.

**Figure 2:**
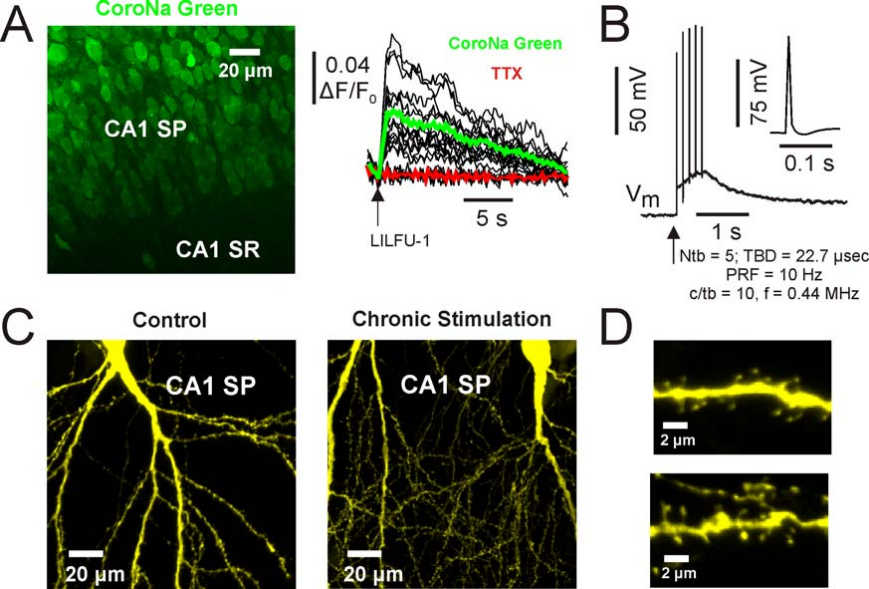
LILFU stimulates sodium transients mediated by voltage-gated sodium channels in hippocampal neurons. (A) Confocal image (*left*) of a slice culture loaded with CoroNa Green AM. Hippocampal regions CA1 *stratum pyramidale* (SP) and *stratum radiatum* (SR) are illustrated. Individual (*black*) and averaged (*color*) Na^+^ transients (*right*) triggered in CA1 pyramidal neuron somas by LILFU-1 under control conditions and in the presence of TTX. (B) Voltage trace of membrane voltage in response to five US tone bursts delivered at a PRF of 10 Hz during whole-cell current clamp recordings of a CA1 pyramidal neuron. (C) Neuronal membrane integrity is preserved following chronic *in vitro* stimulation with LILFU. Confocal images of CA1 pyramidal neurons from hippocampal slice cultures prepared from *thy*-*1*-YFP mice. The images shown are from a control slice culture (*left*) and a slice culture following chronic stimulation (*right*) with LILFU-1 every 8 min for 48 h (360 LILFU-1 stimuli). (D) Similar to (C), but higher magnification images of regions in CA1 SR, which more clearly illustrate the presence of fine membrane structures such as dendritic spines for control (*top*) and chronic LILFU stimulation conditions (*bottom*).

By imaging organotypic hippocampal slice cultures bath-loaded with the Na^+^ indicator CoroNa Green AM [27], we found LILFU-1 triggered Na^+^ transients in hippocampal CA1 pyramidal neurons (ΔF/F_0_  = 0.05±0.006, n = 24, 6 slices; Figure 2A). Addition of the voltage-gated Na+ channel pore blocker tetrodotoxin (TTX; 1 µm), blocked Na^+^ transients evoked by LILFU-1 (Figure 2A). These observations indicate that LILFU-1 increased the Na^+^ conductance in hippocampal neurons by stimulating the opening of voltage-gated Na^+^ channels. We next aimed to determine if LILFU waveforms were also capable of triggering action potentials in CA1 pyramidal neurons. Indeed, we observed single action potentials in response to the delivery of individual LILFU tone bursts during whole-cell current clamp recordings of CA1 pyramidal neurons (n = 4, 4 slices; Figure 2B). We determined however, whole-cell electrophysiological approaches were not very useful in studying the influence of US on neuronal activity since electrode resonances typically cause the loss of whole-cell seals during stimulation with LILFU. Thus, we continued our investigations using standard optophysiological approaches.

Cavitation is one of the best studied non-thermal effects of US on biological tissue [13], [28]. Acoustic cavitation can occur when the intensity of US is sufficient to induce the resonation, expansion, and collapse of gas bodies present in some biological tissue. These microexplosions can influence membrane porosity [12], [13]. Monitored using optical microscopy during LILFU stimulation, we did not observe cavitation in our studies. Additionally, at the acoustic intensities used in our studies, we did not observe other evidence of membrane damage produced by LILFU stimulation. To examine the effect of LILFU on membrane integrity, we chronically stimulated slice cultures prepared from *thy-1*-YFP mice [29] with LILFU-1 every 8 min for 36–48 hours. We observed no difference in the membrane structures of YFP^+^ neurons undergoing chronic stimulation compared to unstimulated controls (n = 9 slices each; Figure 2C, 2D).

### LILFU stimulates voltage-dependent calcium transients in neurons

**Figure 3:**
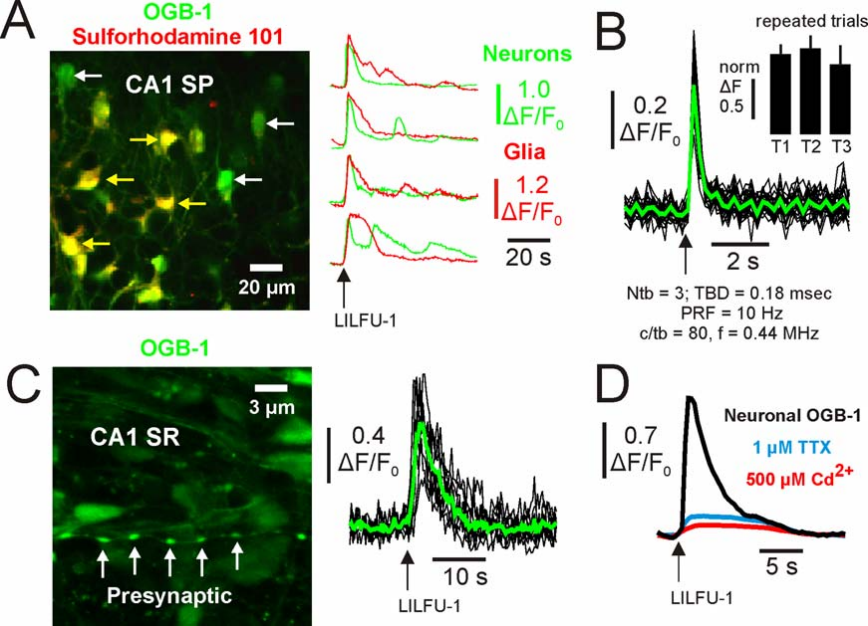
LILFU triggers voltage-dependent somatic and presynaptic Ca^2+^ transients in neurons. (A) Confocal image (*left*) of a slice culture loaded with OGB-1 AM (*green*) to monitor Ca^2+^ activity and Sulforhodamine 101 (*red*) to identify glial cells (*yellow*). Representative LILFU-triggered Ca^2+^ transients observed in the somas of neurons and glial cells are illustrated (*right*). (B) Individual (*black*) and averaged (*green*) Ca^2+^ transients observed in the somas of neurons in response to a brief LILFU waveform. The histogram (*inset*) illustrates trial 1 normalized mean Ca^2+^ transient amplitudes in response to repeated trials of LILFU stimulation (n = 19 cells from 3 slices). (C) Confocal image (*left*) of a slice culture loaded with OGB-1 AM illustrating *en passant* boutons located in CA1 SR. Individual (*black*) and averaged (*green*) presynaptic Ca^2+^ transients (*right*) produced by stimulation with LILFU-1. (D) Averaged somatic Ca^2+^ transients obtained from neurons under control conditions or in the presence of either TTX (n = 36 from 4 slices) or Cd^2+^ (n = 30 from 4 slices) in response to stimulation with LILFU-1.

To determine if LILFU waveforms were capable of activating Ca^2+^ transients, we bath-loaded slice cultures prepared from wild-type mice with the Ca^2+^ indicator Oregon Green 488 BAPTA-1 AM (OGB-1 AM) and Sulforhodamine 101 (to differentiate between neurons and glial cells) as previously described [30]. We found that LILFU-1 activated Ca^2+^ transients in both hippocampal pyramidal neurons (ΔF/F_0_ = 1.14±0.10, n = 61, 10 slices) and glial cells (ΔF/F_0_ = 1.40±0.12, n = 55, 10 slices; Figure 3A and Video S1). Highlighting temporal specificity, stimulation with more brief LILFU waveforms (f = 0.44 MHz, TBD = 0.18 msec, c/tb = 80, PRF = 10 Hz, and Ntb = 3), elicited neuronal Ca^2+^ transients (ΔF/F_0_ = 0.38±0.02, n = 24, 5 slices) with faster kinetics as expected (Figure 3B). In response LILFU stimulation, we observed that Ca^2+^ transients could be repeatedly obtained from neurons across multiple LILFU stimulation trials (Figure 3B). While we primarily focused on small regions of interest during stimulation, when we imaged large fields of view we observed that approximately 30% of the neurons respond to LILFU-1. Stimulation with LILFU-1 also induced presynaptic Ca^2+^ transients in *en passant* boutons located in CA1 SR (ΔF/F_0_ = 0.76±0.07, n = 31 from 4 slices; Figure 3C). Addition of Cd^2+^ (500 µM) nearly abolished OGB-1 signals in response to LILFU-1, indicating Ca^2+^ transients triggered by LILFU are primarily mediated by voltage-gated Ca^2+^ channels (Figure 3D). Likewise, the addition of TTX blocked ∼85% of the OGB-1 signal produced by LILFU-1 (Figure 3D). Residual Ca^2+^ transients not blocked by Cd^2+^ or TTX are likely to involve other hippocampal neuron Ca^2+^ sources such as NMDA or TRPC1 receptors, which is consistent with both channels possessing mechanosensitive properties [31], [32] and being expressed in hippocampal neurons.

**Figure 4:**
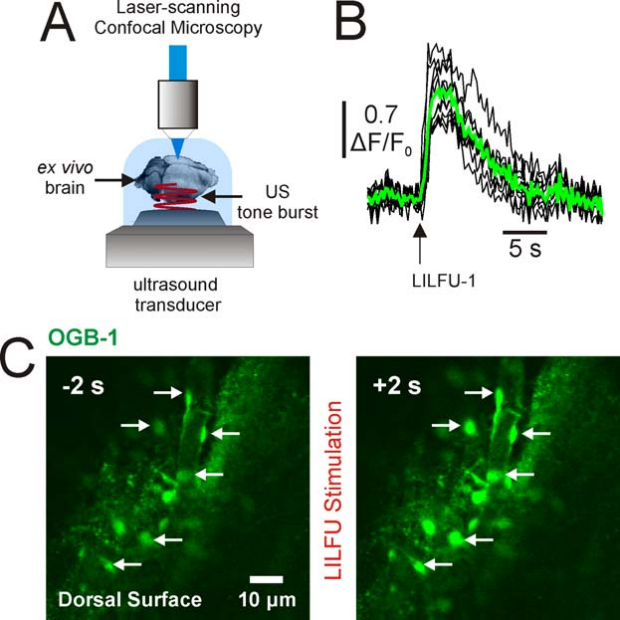
LILFU waveforms transmitted through whole brains are capable of stimulating calcium transients. (A) Illustration of basic experimental procedure we developed to transmit LILFU waveforms through whole *ex vivo* brains prepared from adult wild-type mice and bath-loaded with OGB-1 AM. As depicted, LILFU waveforms were transmitted from the ventral surface of the brain through the tissue to the dorsal surface where we performed confocal imaging. (B) Individual (*black*) and averaged (*green*) Ca^2+^ transients observed in the somas of cells on the dorsal surface of an *ex vivo* brain in response to stimulation with LILFU-1, which was transmitted through the brain from the ventral surface. (C) Confocal images illustrating OGB-1 loaded cells on the dorsal surface of the brain. The image on *left* illustrates cells during baseline, while the image on the *right* illustrates cells two-seconds after stimulation with LILFU-1 ensued.

We were able to observe Ca^2+^ transients in response to pulsed US even when transducers were placed as far as 45 mm away from slices (n =  5; data not shown). Similar to water and aqueous buffers, soft biological tissues (including brain) have relatively low acoustic absorption coefficients. Therefore, we sought to determine if LILFU propagated through whole brain tissue was also capable of stimulating neuronal activity. We imaged OGB-1 signals on the dorsal superficial surface of *ex vivo* brains (n = 3) obtained from wild-type adult mice while transmitting LILFU waveforms through their ventral surfaces (Figure 4A). In these *ex vivo* brain preparations, we observed Ca^2+^ transients similar to those observed in thinner and less intact slice culture preparations in response to stimulation with LILFU (Figure 4B, 4C).

### LILFU triggers SNARE-mediated synaptic vesicle exocytosis and synaptic transmission

**Figure 5:**
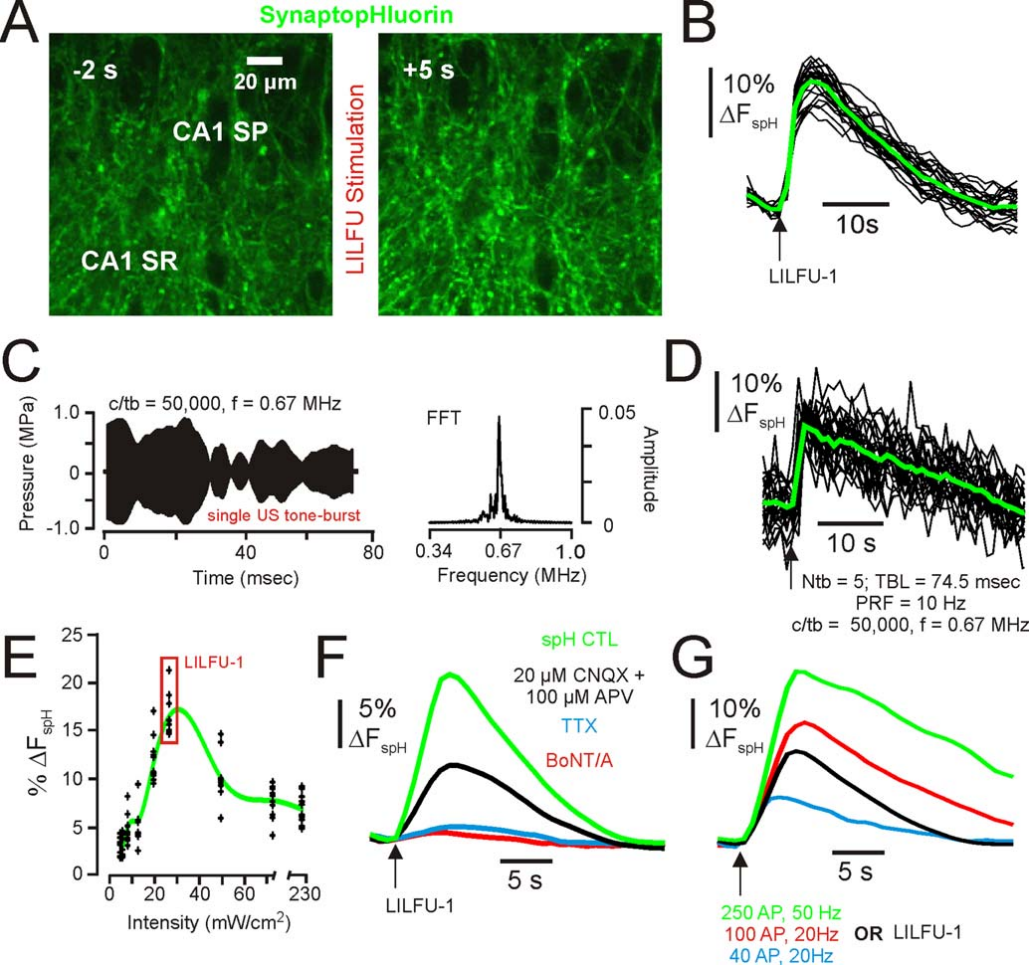
LILFU stimulates SNARE-mediated synaptic vesicle exocytosis and central synaptic transmission. (A) Confocal images illustrating spH signals obtained before (*left*) and during (*right*) stimulation with LILFU-1. (B) Individual (*black*) and averaged (*green*) spH signals typically obtained in response to stimulation with LILFU-1. (C) Acoustic pressure wave (*left*) produced by a single LILFU tone burst consisting of 50,000 acoustic cycles at f = 0.67 MHz and FFT of LILFU tone burst (*right*). (D) Individual (*black*) and averaged (*green*) spH signals obtained in response to stimulation with the LILFU tone burst shown in (C) delivered at a PRF = 10 Hz for 0.5 s to produce Np = 5. (E) Histogram of spH responses obtained as a function of acoustic intensity. Responses from individual experiments are indicated by *black crosses* while the average response is indicated by the *green line*. (F) Averaged spH signals illustrating the effect of CNQX+APV (n = 84 from 4 slices), TTX (n = 108 from 4 slices), or BoNT/A (n = 60 from 4 slices) on synaptic vesicle exocytosis induced by LILFU-1. (G) Averaged spH signals obtained from buttons in response to field stimulation of Schaffer collaterals with 250 AP, 50 Hz (n = 48), 100 AP, 20 Hz (n = 63), 40 AP, 20 Hz (n = 51), or by LILFU-1 (n = 148).

**Figure 6:**
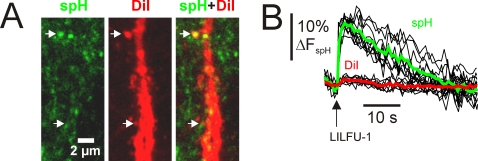
Influence of LILFU on putative excitatory hippocampal CA3-CA1 synapses. (*A*) Confocal images illustrating spH expression in CA1 SR (*left*) and an apical dendritic branch of a CA1 pyramidal neuron, which was labeled with DiI using a DiOlistic labeling technique (*middle*). The two-channel confocal image (*right*) illustrates putative excitatory synapses indicated by apposition of spH^+^ puncta and dendritic spines. (*B*) Individual (*black*), mean spH (*green*), and mean DiI (*red*) signals obtained from terminals impinging on dendritic spines in response to stimulation with LILFU-1.

To investigate the influence of LILFU on synaptic transmission we focused on studying a well-characterized synapse in the mammalian central nervous system, the hippocampal CA3-CA1 synapse. We transmitted LILFU waveforms through hippocampal slice cultures prepared from *thy-1*-synaptopHluorin (spH) mice [33]. The pH-dependent optical probe of synaptic vesicle exocytosis spH reflects neurotransmitter release through an increase in fluorescence when protons are released from synaptic vesicles during fusion [34]. Transmission of LILFU-1 through slices triggered synaptic vesicle exocytosis producing a ΔF_spH_ of 18.52±2.2% at individual release sites (n = 148 from 15 slices) in CA1 *stratum radiatum*, which primarily represent CA3-CA1 synapses (Figures 5A, 5B and Video S2). We identified several other LILFU waveforms, which were also effective at triggering synaptic vesicle release (Table S1). For example, a LILFU waveform composed of different US tone bursts (f = 0.67 MHz, TBD = 74.5 msec, c/tb = 50,000; Figure 5C) delivered at PRF = 10 Hz with Ntb = 5 also stimulated synaptic vesicle release (ΔF_spH_ = 12.86±2.6%, n = 74 from 6 slices; Figure 5D). Figure 5E illustrates spH responses obtained as a function of acoustic intensity across several different LILFU waveforms used in this study. To more specifically examine excitatory CA3-CA1 hippocampal synapses, we implemented a DiOlistic labeling approach [35] to visualize dendritic spines on CA1 apical dendrites in *thy-1*-spH slices cultures. Indeed, LILFU-1 stimulated synaptic vesicle release in this population of spine synapses (Figure 6).

Hyperosmotic shock produced by application of sucrose to hippocampal synapses is capable of stimulating the release of a small pool of primed synaptic vesicles (∼10 vesicles) in a Ca^2+^-independent manner and is thought to occur from mechanical processes [36]. Due to the nature of mechanical energy conferred by acoustic waves, we questioned whether some part of the synaptic vesicle release we observed in response to LILFU might be due to mechanical interactions on vesicle release machinery or between the lipid bilayers of active zones and synaptic vesicles. Since hypertonic sucrose application is still capable of triggering neurotransmitter release at hippocampal synapses lacking the SNARE-protein SNAP-25 [37], we aimed to determine if LILFU-1 was capable of stimulating neurotransmitter release after cleaving SNAP-25 by treating slice cultures with botulinum neurotoxin type-A (BoNT/A; 24–36 h). Indicating that pulsed US-induced exocytosis is SNARE-mediated and not likely due to mechanisms similar to those produced by hyperosmotic shock, treatment of slice cultures with BoNT/A nearly abolished spH responses produced by LILFU-1 stimulation (Figure 5F).

Addition of TTX almost completely blocked vesicular release in response to LILFU-1 highlighting the importance of Na^+^ conductance and action potentials in LILFU-triggered synaptic vesicle release (Figure 5F). Blocking excitatory network activity with CNQX (20 µM) and APV (100 µM) reduced the ΔF_spH_ by ∼50% compared to controls indicating that LILFU stimulates synaptic transmission (network activity) and not merely exocytosis (Figure 5F). Interestingly, the kinetics and amplitudes of LILFU-triggered spH signals were nearly identical to those obtained in response to electrical stimulation of CA3 Schaffer collaterals using monopolar electrodes (Figure 5G), as well as those spH responses previously reported [33], [38]. Since spH typically produces a ΔF of ∼1–2% per released vesicle [38], [39], we estimated LILFU-1 to stimulate the release of ∼15 vesicles per release site.

## Discussion

In this study we tested whether LILFU was capable of directly stimulating the activity of neurons in the central nervous system. We made several novel observations in our study. From a mechanistic view, we observed that US stimulates neuronal activity at least partially by triggering voltage-gated Na^+^ transients and voltage-dependent Ca^2+^ transients. We further observed the US-induced changes in neuronal activity were sufficient to trigger SNARE-mediated synaptic vesicle exocytosis and synaptic transmission at central synapses thereby driving network activity.

The mechanisms underlying US activation of voltage-sensitive channels in neurons are presently unknown. We postulate however the mechanical nature of US and its interactions with neuronal membranes leads to the opening of mechanically sensitive voltage-gated channels. Supporting this hypothesis, we observed that TTX a voltage-gated Na^+^ channel pore-blocker attenuated LILFU-triggered Na^+^ transients. Further, many voltage-gated Na^+^ channels (i.e. NaV 1.2, 1.4, 1.5, and 1.6) are known to possess varying degrees of mechanical sensitivity [16], [17]. The addition of TTX also blocked a large portion of LILFU-induced Ca^2+^ transients indicating the primary action of LILFU may be on voltage-gated Na^+^ channels. However, the addition of Cd^2+^ further reduced LILFU-activated Ca^2+^ transients, which suggests at least some voltage-gated Ca^2+^ channels may be sensitive to LILFU. Indeed, L-type, N-type, T-type, and P-type Ca^2+^ channels have been shown to be mechanically sensitive under various conditions [16], [17].

Further studies are required to identify which ion channels are sensitive to US, as well as to characterize how these channels respond to US as a function of acoustic intensity. By imaging large fields of view and monitoring the responses from large populations of neurons, we observed that LILFU-1 stimulated activity in ∼30% of the neurons in a given field. These observations raise several interesting issues. We question for instance whether neurons, which have been recently active, are less susceptible to US stimulation. In other words, the kinetic states of a neuron's ion channels may shape how responsive a given cell is to US stimulation. It could also be the case that recently active neurons are more responsive to US stimulation. We are currently in the process of investigating these issues. The individual properties of US waveforms (peak and temporal average intensity, tone burst/pulse duration, pulse repetition frequency, etc.) will also likely determine how effective a given waveform is at stimulating neuronal activity. With respect to acoustic intensity for example, we observed that US waveforms having moderate intensities were more robust in triggering synaptic transmission compared to US waveforms possessing lower or higher intensities. Future studies investigating the influence of US on neuronal activity should consider interactions among waveform parameters such as tone-burst duration (pulse length), pulse repetition frequency, exposure time, acoustic frequency, and acoustic intensity. Understanding how waveform characteristics contribute to the actions of US on neuronal activity will be an important issue to resolve. One particularly interesting question is can LILFU be used in a molecularly specific manner–perhaps by inducing protein specific resonances using an optimal acoustic frequency or particular LILFU waveform?

### Potential biohazardous effects of US

Having a long and proven safety record, US is widely used for diagnostic medical imaging, as well as in an array of noninvasive therapies [13]. Ultrasound is however quite capable of destroying biological tissues, so when employing US to stimulate neuronal activity the potential for biohazardous effects must be carefully considered. Many of the hazards associated with US stem from its ability to induce large thermal fluctuations and/or cavitational damage in soft tissues. Although many groups have previously demonstrated an effect of US on neuronal activity [3], [4], [18]–[24], these results are unique in that we found US is capable of stimulating neuronal activity at lower acoustic intensities than those previously reported. Some groups have utilized acoustic intensities as low as 1 W/cm^2^ to modulate neuronal activity in hippocampal brain slices [19], whereas other groups have used intensities exceeding 1000 W/cm^2^ to trigger peripheral pain sensations in humans [3]. In this study we implemented a range of acoustic intensities where the nonthermal effects of US have been well documented in other tissues (30–500 mW/cm^2^) [5], [11]–[13]. Further, the US intensities we found sufficient for stimulating neuronal activity are below the output power limits set by the United States Food and Drug Administration for diagnostic imaging.

Due to the lack of gas bodies in most soft tissues including brain [13], we do not expect cavitation to pose significant problems when using LILFU to stimulate brain activity *in vivo.* In most soft tissues, cavitation rarely induces damage at pressures <40 MPa (except for lung, intestinal, and cardiac tissues in which cavitational damage can occur at pressures ∼2 MPa due to the presence of naturally occurring gas bodies) [13]. The peak rarefactional pressure used in our studies was <1 MPa. At the US power levels we studied, cavitational damage was not induced in hippocampal slice cultures. Besides the potential biohazards of acute US transmission into brain tissue, the possibility for damage arising from repeated, long-term US exposure needs to be evaluated. Few studies have examined the effects of chronic US administration on brain function. We found that chronic LILFU stimulation (36–48 h) did not alter the fine structure of neuronal membranes. Demonstrating the need for caution however, a recent study reported that repeated US exposure is capable of producing some disruption of neuronal migration in the cortex of developing mouse embryos [40].

### The effects of US on molecular signal transduction pathways

While we have studied the actions of US on neuronal activity by monitoring ionic conductance and synaptic vesicle exocytosis, we recognize US may influence signaling molecules capable of influencing neuronal function. In other tissues, the activity of several signaling molecules also present in neuronal tissues are known to be influenced by US. For example, low-intensity pulsed US stimulates TGF-β signaling, which triggers the differentiation of human mesynchymal stem cells into chondrocytes [41]. Low-intensity pulsed US has also been shown to stimulate the production of bFGF, TGF-β, BMP-7, VEGF, and IGF-1 [42]–[45]. Certainly bFGF, TGF-β, BMP-7, VEGF, and IGF-1 have differential yet significant effects on the nervous system by affecting processes involved in synaptic transmission, neuronal growth/survival [46], [47], cell fate specification, tissue patterning, axon guidance in the nervous system [48], and angiogenesis in the brain [49]. Moreover, VEGF [49], [50], TGF-β [51], [52], and bFGF [46] are neuroprotective against hypoxic-ischemic injury and neurodegeneration. These observations prompt the intriguing question of whether it is possible for US to trigger these pathways in the brain or the production and secretion of growth factors such as brain-derived neurotrophic factor, neurotrophin-3, or nerve growth factor.

Additional actions on conserved cell signaling pathways further support explorations into the use of US as a neuromodulation tool. NF-κB is known to regulate neuronal survival and plasticity [53]. Integrin-linked kinase (ILK) and Akt are known to be important signals in establishing neuronal polarity [54]. The PI3K-Akt signaling pathway is capable of blocking cell death and promoting cell survival of many neuronal cell types [55]. Ultrasound induces cyclooxegynase-2 expression in human chondrocytes by activating the integrin/ILK/Akt/NF-κB/ and p300 signaling pathway [56], while in murine osteoblasts US stimulates COX-2 expression via the integrin/FAK/PI3K/Akt and ERK signaling pathway [57]. It should be determined if US is also capable of stimulating ILK, PI3K, Akt, and or NF-κB signaling in neurons as these signaling molecules may become important targets for future ultrasonic neuromodulation strategies.

### Feasibility of delivering LILFU to intact nervous systems and brains for neuromodulation

As a tool for modulating neuronal function, US has been studied and considered across a range of uses from thermal ablation of nervous tissues to its ability to produce sensory perceptions [6], [9], [10]. Gavrilov and colleagues (1976) were the first to show that US is capable of activating both superficial and deep peripheral nerve structures in humans, which lead to different thermal, tactile, and pain sensations. In these studies however, US was only transmitted through soft tissues such as the skin to stimulate neuronal activity. Whether US will be effective in the noninvasive transcranial regulation of neuronal circuits in the intact nervous system remains to be determined.

Transcranial ultrasonography of the basilar artery has been shown to trigger auditory sensations in human subjects [58]. Other studies have reported similar observations in animals during delivery of transcranial US and at least one underlying mechanism is thought to involve the direct stimulation of auditory nerve fibers by US [10]. Collectively, these observations demonstrate transcranial US is capable of evoking sensory stimuli even in humans. Despite these exciting observations, the skull is a major obstacle when considering the transmission of US into intact brains for neurostimulation purposes. The skull reflects, refracts, absorbs, and diffracts US fields. Acoustic impedance mismatches between the skin, skull, and skull-brain interfaces also present a challenge for transmitting US through the skull into the intact brain. The frequency of US we chose for the construction of LILFU waveforms (0.44–0.67 MHz) represents a range where optimal gains have been previously reported between transcranial US transmission and brain absorption. Based on modeling data of transmission and attenuation coefficients, as well as experimental data examining the transmission of US through *ex vivo* human skulls, the optimal gain for the transcranial US transmission and brain absorption is between 0.60 and 0.70 MHz [25], [26]. Based on our observations and the findings of others, it is likely that LILFU fields can be transmitted through skulls into the intact brain for gross neurostimulation purposes similar to methods using rTMS. In order to achieve targeted neurostimulation however, it will be necessary to focus LILFU fields.

It is possible to focus US fields using a variety of approaches. Pulsed US (<1 MHz) can be focused through human skulls to points within 1 mm of intended loci using phased US transducer arrays [6], [8], [59]. Based on observations reported in studies designed to investigate US field focusing through human skulls [6], [8], [59], US may be able to confer a spatial resolution similar to those achieved by currently implemented neuromodulation strategies such as vagal nerve stimulation and DBS, which have been shown to possess high therapeutic value [1], [60]. Before the feasibility of using focused LILFU for targeted neurostimulation purposes can be properly determined, future studies must directly address how focused US fields influence the activity of neuronal populations *in vivo*.

### Conclusions

Our observations demonstrate that LILFU can be used to remotely stimulate the activity of central nervous system neurons and circuits *in vitro*. We have provided the first direct evidence that US modulates the ionic conductance of neurons and astrocytes to increase cellular activity and synaptic transmission in a manner sufficient to stimulate neuronal circuits. Several issues need to be resolved before the full potential of US in controlling neuronal activity can be realized. Since US is capable of being focused through the human skull however, one tantalizing possibility is that LILFU may permit deep-brain stimulation without the need for surgically implanted devices or other invasive procedures.

## Materials and Methods

### Preparation of slice cultures and *ex vivo* brains

All procedures involving mice were conducted in accordance with federal guidelines and protocols approved by the Institutional Animal Care and Use Committee at Arizona State University. Hippocampal slice cultures were prepared from postnatal day 7–8 *thy*-*1*-spH, *thy*-*1*-YFP, or wild-type mice similar to previously described methods [61]. Briefly, transverse hippocampal slices (∼400 µm thick) were made using a wire slicer (MX-TS, Siskiyou, Inc., Grants Pass, Oregon, USA) and maintained *in vitro* on Millicell-CM filter inserts (PICMORG50, Millipore, Bedford, MA) in a 36°C, 5% CO_2_, humidified (99%) incubator. Slices were used for experiments between 7 and 12 days *in vitro*. In some experiments to cleave SNARE-proteins, BoNT/A (250 ng/mL) was added to the slice culture media 24–36 h prior to use.

We prepared *ex vivo* brains using the following approach. Following CO_2_ inhalation, wild-type mice were rapidly decapitated and their brains were removed. The dura was carefully removed and the brains were then placed in ice-cold artificial CSF (aCSF) containing (in mM) 83 NaCl, 2.5 KCl, 3.3 MgSO_4_, 1 NaH_2_PO_4_, 26.2 NaHCO_3_, 22 glucose, 72 sucrose, and 0.5 CaCl_2_, and equilibrated with 95% O_2_/5% CO_2_. Brains were allowed to recover for 5 min in the ice-cold aCSF before recovering for ∼20 min at 37°C. Following this recovery period, *ex vivo* brains were bulk loaded with OGB-1 AM (Invitrogen, Carlsbad, California, USA).

### Loading of slice cultures and *ex vivo* brains with fluorescent ion indicators

In order to load slice cultures prepared from wild-type mice with CoroNa Green AM (Invitrogen, Carlsbad, California, USA), 5 µL 20% Pluronic F-127 in DMSO (Invitrogen) was added to a 50 µg vial of CoroNa Green AM. The dye solution was then vortexed for 15 min before adding 100 µL culture medium. We then added 5 µL of the dye-containing solution to 1 mL culture medium underneath culture inserts, as well as adding 5 µL to the surface of slices. Following a 10 min incubation time at 36°C, slices were washed three times with slice culture medium, allowed to recover an additional 10 min, and then used for experiments. To load slice cultures with OGB-1 AM, we added 2 µL 20% Pluronic F-127 in DMSO and 8 µL DMSO to a 50 µg vial of OGB-1 AM. The dye-containing solution was then vortexed for 30 M before adding 90 µL culture media. We next added 20 µL of this dye-containing solution to 3 mL culture medium and incubated slices in this solution for 30–40 min at 37°C. Slices were washed three times with slice culture medium, then loaded with sulforhodamine 101 (Invitrogen; 10 µM in slice culture medium for 15 min) or allowed to recover for 30 min prior to an experiment. To load *ex vivo* brains with OGB-1 AM we used a procedure similar to above, but substituted the slice culture medium for dissection aCSF (see above)–we added 60 µL of the dye-containing solution to 9 mL dissection aCSF. Brains were loaded for 30 min at room temperature then rinsed three times and allowed to recover for an additional 30 min in dissection aCSF at room temperature before use.

### Confocal imaging and whole-cell patch-clamp recordings

Slice cultures or whole *ex vivo* brains were transferred to recording chambers containing recording aCSF (in mM) 136 NaCl, 2.5 KCl, 1.3 MgSO_4_, 10 HEPES, 10 glucose, and 2.5 CaCl_2_ , pH 7.4 at room temperature. Recording chambers were affixed above US transducers on a custom built-stage on an Olympus Fluoview FV-300 laser-scanning confocal microscope (Olympus America, Inc., Center Valley, Pennsylvania, USA). Excitation of spH, OGB-1 AM, and CoroNa Green AM was performed using the 488 nm laser-line of an argon laser and in some experiments DiI was excited using a 546 nm HeNe laser. Time-series images were acquired using 20×(0.5 NA) or 40×(0.8 NA) Olympus UMPlanFL water-immersion lens.

Slice recording chambers consisted of culture inserts placed inside an aCSF reservoir held in place with either vacuum grease on the silicon face of the transducer. This approach produced ∼4.5 mm standoff distance between the face of the transducer and the imaging plane on the surface of slices. In a subset of experiments, slice cultures (n = 5) were mounted near the top of an aCSF column in a 500 mL beaker containing immersed US transducers, which were affixed to the bottom beakers to provide a 45 mm standoff distance. To image *ex vivo* brains, the ventral surface of whole *ex vivo* brains were glued to the bottom of polystyrene 6-well plates using superglue, which were filled with aCSF and mounted above US transducers using ultrasonic coupling gel. Confocal imaging of OGB-1 fluorescence was conducted on the superficial dorsal surface of *ex vivo* brains during transmission of LILFU waveforms from the ventral surface of the brain.

In a subset of experiments we performed whole-cell current clamp recordings from visually identified CA1 pyramidal neurons using standard approaches. Briefly, patch electrode pipettes filled with an intracellular solution containing (in mM) 130 KCl, 10 Na-HEPES, 10 Di-Tris-P-creatine, 0.2 EGTA, 3 Mg-ATP, and 0.5 Na-GTP, 280–290 mOsm, pH 7.2; the final resistance of these unpolished patch electrodes was 5–7 MΩ. Current clamp recordings were performed using a MultiClamp 700B patch-clamp amplifier with pCLAMP 10 software (Molecular Devices, Sunnyvale, California, USA). Following 5–10 min of whole-cell access, changes in membrane voltage were recorded in response to stimulation with LILFU waveforms.

### Generation and characterization of LILFU waveforms

In our studies we used custom built PZT ultrasound transducers (d = 35 mm) having a single quarter-wave matching layer, a center frequency of 0.53 MHz, and a −6 dB fractional bandwidth of 65% with two peaks (0.44 MHz, 0.66 MHz). LILFU waveforms used as stimuli were generated by repeating pulse trains of US tone bursts at a pulse repetition frequency until a desired number of tone bursts had been generated (Figure 1B). Ultrasound tone bursts were generated by trains of square waves (0.2 µsec) with variable amplitudes (Table S1) using an Agilent 33220A function generator. To produce final plate voltages delivered to transducers, square waves were further amplified (50 dB gain) using an ENI 240L RF amplifier. Square waves were delivered between 0.44–0.67 MHz depending on the acoustic frequency desired, while the number of square waves driving each US tone burst equaled the number of acoustic cycles desired for a given US tone burst. Each US tone burst (pulse) contained between 1 and 50,000 acoustic cycles depending on the LILFU waveform generated. US tone bursts (Figure 1B) were repeated at a pulse repetition frequency by triggering the above referenced function generator with a second Agilent 33220A function generator. Pulse repetition frequencies were either a constant frequency or a swept waveform. Our primary LILFU waveform (LILFU-1) had the following properties: f = 0.44 MHz, TBD = 22.7 µs, c/tb = 10, PRF = 5 sec sweep 0–100 Hz, and Ntb = 250.

To characterize LILFU power levels, we recorded voltage waveforms produced by US pressure waves using a hydrophone (HNR 500, Onda Corporation, Sunnyvale, California, USA) and an Agilent DSO6012A 100 MHz digital oscilloscope (Agilent Technologies, Inc., Santa Clara, California, USA). To confirm transducers were operating at the intended acoustic frequency, we performed an FFT on hydrophone voltage traces recorded in response to US tone bursts. All pressure waves produced by LILFU waveforms were measured at points corresponding to tissue positions in the actual recording chambers by positioning the hydrophone face using a *xyz* micromanipulator (MP-225, Novato, CA, USA) mounted on the vibration isolation table attached to the microscope stage (Figure S1). The position of slices in recording chambers was held consistent across experiments. We measured acoustic intensities with and without slices in the recording chamber and found no effect of the presence of a slice on the acoustic waveform. The acoustic pressure and ultrasonic intensities (I_PA_ and I_TA_) were calculated using published equations and technical standards established by the American Institute of Ultrasound in Medicine and the National Electrical Manufacturers Association [62].

### Data analysis

Confocal images were analyzed offline using *ImageJ* (http://rsb.info.nih.gov/ij/) or the Olympus Fluoview 5.0 software. We express changes in spH fluorescence as a percent change from baseline fluorescence levels. For OGB-1 and CoroNa Green signals, we calculated ΔF/F_0_ using standard approaches where ΔF = F−F_0_. LILFU waveforms and electrophysiological analyses were performed offline using *Igor Pro* (WaveMetrics, Lake Oswego, Oregon, USA). Data shown are mean±S.E.M.

## Supporting Information

Figure S1 Characterization and operation of PZT transducers. Illustration of experimental setup used to operate PZT transducers and transmit LILFU waveforms through neuronal tissue. For measuring PZT properties, as well as the pressure waves produced by US tone bursts, we used a calibrated hydrophone. To investigate the influence of LILFU on neuronal activity, we transmitted LILFU waveforms through a column of aCSF into hippocampal slice cultures while simultaneously performing confocal microscopy (see *Materials and Methods* for further details).
(2.80 MB TIF)

Table S1 (0.05 MB DOC)

Video S1 The video illustrates a time-lapsed series of confocal images obtained from an organotypic slice culture prepared from a wild-type mouse, which was bath-loaded with OGB-1 AM. Hippocampal CA1 *stratum pyramidale* is indicated. The appearance of *red* stim indicates the delivery of LILFU-1. As indicated by the increase in OGB-1 fluorescence intensity, Ca2+ transients were triggered in response to stimulation with LILFU-1.
(7.87 MB AVI)

Video S2 The video illustrates a time-lapsed series of confocal images obtained from a thy-1-spH organotypic slice culture. Hippocampal CA1 *stratum pyramidale* is in the upper left region of the movie with the proximal portion of *stratum radiatum* emerging towards the lower right quadrant of the movie. The appearance of *red* stim indicates the delivery of LILFU-1. As indicated by the increase in spH fluorescence intensity, the induction of vesicle release in response to LILFU can be clearly resolved at individual buttons.
(7.56 MB AVI)
