# Supplementary figures and images for: Remote Excitation of Neuronal Circuits Using Low-Intensity, Low-Frequency Ultrasound
Source: PLoS One. 2008 Oct 29;3(10):e3511. doi: 10.1371/journal.pone.0003511 (PMC2568804; doi:10.1371/journal.pone.0003511)

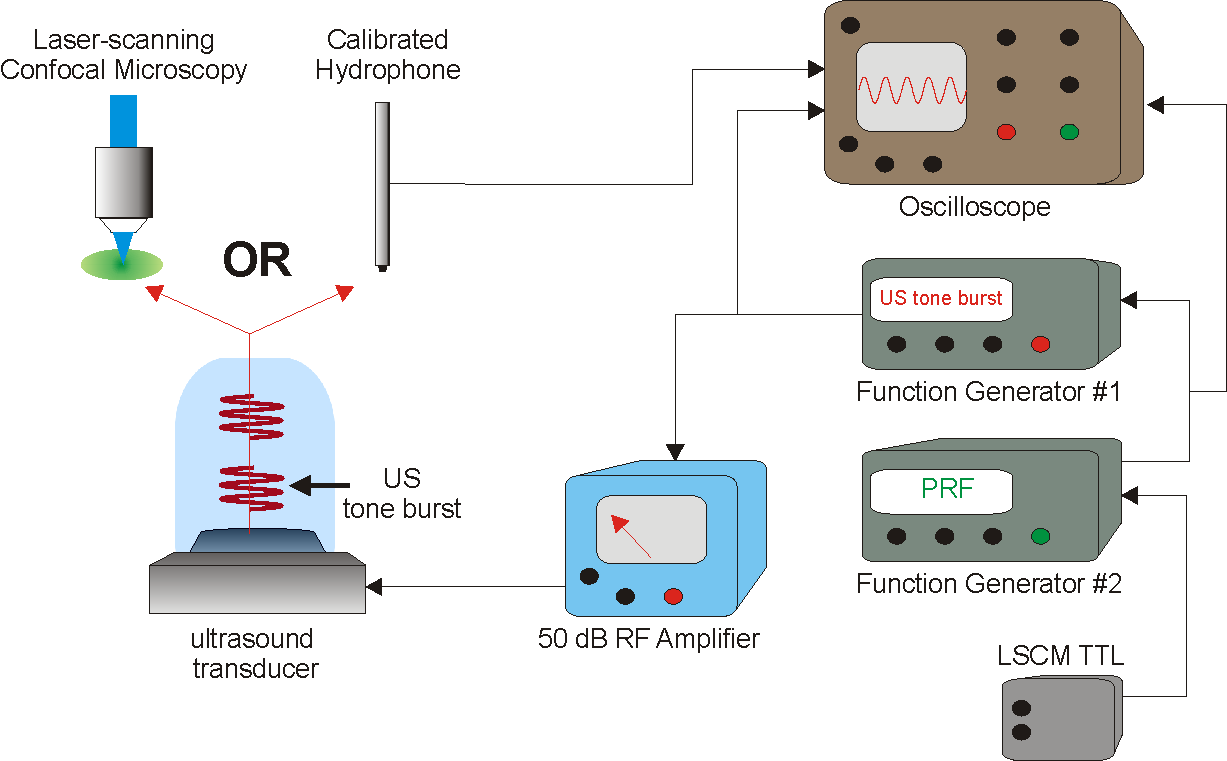

Supplement: Figure S1 — Characterization and operation of PZT transducers. Illustration of experimental setup used to operate PZT transducers and transmit LILFU waveforms through neuronal tissue. For measuring PZT properties, as well as the pressure waves produced by US tone bursts, we used a calibrated hydrophone. To investigate the influence of LILFU on neuronal activity, we transmitted LILFU waveforms through a column of aCSF into hippocampal slice cultures while simultaneously performing confocal microscopy (see Materials and Methods for further details). (2.80 MB TIF) [file pone.0003511.s001.tif]
