# Supplementary material for: Remote Excitation of Neuronal Circuits Using Low-Intensity, Low-Frequency Ultrasound
Source: PLoS One. 2008 Oct 29;3(10):e3511. doi: 10.1371/journal.pone.0003511 (PMC2568804; doi:10.1371/journal.pone.0003511)
Supplement: Table S1 — (0.05 MB DOC) [file pone.0003511.s002.doc]

**Table S1**

Summary of the effectiveness of LILFU waveforms in stimulating synaptic vesicle release.

| **TBL** | **c/tb** | **f (MHz)** | **PRF** | **Ntb** | **p-p sq. wave amplitude** | **spH response** |
| --- | --- | --- | --- | --- | --- | --- |
| 22.7 μs | 10 | 0.44 | 5 s sweep  0 - 100 Hz | 250 | 500 mV | + |
| 74.5 ms | 50,000 | 0.67 | 10 Hz | 5 | 150 mV | + |
| 74.5 ms | 50,000 | 0.67 | 10 Hz | 150 | 100 mV | + |
| 22.7 μs | 10 | 0.44 | 5 s sweep  0 - 100 Hz | 250 | 100 mV | - |
| 2.27 μs | 1 | 0.44 | 5 s sweep  0 - 100 Hz | 250 | 500 mV | - |
| 11.35 μs | 5 | 0.44 | 5 s sweep  0 - 100 Hz | 250 | 500 mV | + |
| 11.35 μs | 5 | 0.44 | 5 s sweep  0 - 100 Hz | 250 | 100 mV | - |
| 2.27 μs | 1 | 0.44 | 20 Hz | 100 | 500 mV | - |
| 22.7 μs | 10 | 0.44 | 20 Hz | 100 | 500 mV | + |
| 22.7 μs | 10 | 0.44 | 250 Hz | 250 | 500 mV | - |
| 22.7 μs | 10 | 0.44 | 10 s sweep  0 - 100 Hz | 500 | 500 mV | + |
| 22.7 μs | 10 | 0.44 | 15 s sweep  0 - 100 Hz | 750 | 500 mV | + |
| 113.5 μs | 50 | 0.44 | 5 s sweep  0 - 10 Hz | 25 | 500 mV | - |
| 113.5 μs | 50 | 0.44 | 5 s sweep  0 - 200 Hz | 500 | 500 mV | + |
| 113.5 μs | 50 | 0.44 | 5 s sweep  0 - 100 Hz | 250 | 500 mV | + |
| 170.25 μs | 75 | 0.44 | 5 s sweep  0 - 100 Hz | 250 | 500 mV | + |
| 227.0 μs | 100 | 0.44 | 5 s sweep  0 - 100 Hz | 250 | 500 mV | + |
| 22.7 μs | 10 | 0.44 | 50 Hz | 250 | 500 mV | - |

LILFU-1
